# From principles to practice: A code of conduct for the human-centered and ethical development of immersive technologies

**DOI:** 10.12688/openreseurope.22230.1

**Published:** 2026-02-03

**Authors:** XR4HUMAN (The Equitable, Inclusive, and Human-Centered XR), Rosemarie De La Cruz Bernabe, Rigmor C. Baraas, Shereen Cox, Lene A. Hagen

**Affiliations:** 1 Centre for Medical Ethics, Institute of Health and Society, Faculty of Medicine, University of Oslo, Oslo, Norway; 2 National Centre for Optics, Vision and Eye Care, University of South-Eastern Norway, Kongsberg, Buskerud, Norway

**Keywords:** eXtended reality, Immersive technologies, Ethics, Virtual reality, Virtual worlds, Code of Conduct, Augmented reality, Mixed reality

## Abstract

The XR4Human Code of Conduct was developed over a 12-month period using a participatory co-design approach that combined multi-disciplinary expert workshops with members of the consortium followed by stakeholder and public consultations. The process was intentionally iterative. Drafting, review, and revision were guided by reflective equilibrium to reconcile empirical inputs from the project deliverables, normative principles, and practical constraints. The final Code is organised into nine domains that consolidate core principles relevant to the responsible design, development, and deployment of XR systems. These principles are made more concrete through seven articles that specify actionable obligations. To support real-world adoption, the Code is operationalised via an Ethical Impact Assessment and a companion Compliance Checklist aligned to the core principles and articles.

## Disclaimer

The views expressed in this article are those of the authors. Publication in Open Research Europe does not imply endorsement of the European Commission.

## Introduction

Immersive technologies aka eXtended reality (XR) is fast becoming a highly relevant technology with a wide range of applications spanning different industries. Concurrent with the development and the applications are various ethical and legal challenges. The Equitable, Inclusive, and Human-Centered XR project ( XR4Human) had a goal to map various legal, ethical and interoperability challenges of XR technologies and propose guidance for developers and users of XR embedded in human centred and ethics by design principles. To achieve this, researchers in the project employed within seven work packages (WP) over a period of three years deep dived into XR literature and conducted stakeholder surveys and interviews to clearly elucidate and articulate ethical, legal and technological issues. These were published on the XR4human website and in peer reviewed journals. Reports include mapping of a) ethical issues in XR (WP 2), b) regulatory and governance issues (WP 3), c) existing and emerging interoperability and seamless integration of XR (WP4), and d) challenges and presentation of ethical, legal regulatory and technical interoperability solutions. ( XR4HUMAN, n.d.). The combined recommendations of these reports needed to be translated into a normative framework that could be used by XR industry stakeholders from idea to development and deployment. To this end, the normative output of the project is a Code of Conduct (CoC) for human-centered and ethical development of immersive technologies ( Bernabe *et al.*, 2025).

Additionally, it is important that the CoC is not a document that provides superficial guidance but one which is practical for seamless adoption by developers and users. This approach aligns with translational ethics which bridges theoretical, empirical and practical aspects of ethics i.e. ensuring the actual application of ethical principles to real world situations ( Bærøe, 2014). One way to bridge the gap between theory and practice is the use of impact assessments. Impact assessments have become commonplace for effecting standards in various industries including artificial intelligence, risk management and privacy ( Trilateral Research, 2020; Wernick, 2024). The approach is to consider relevant principles, values or issues of an area of impact drawing from various sources, then create questions that would cause one to reflect on the impact of the idea/work in progress ( Wright, 2011). In the context of ethical impact assessment of technology, David Wright notes:

“an ethical impact assessment is needed of new and emerging technologies because technologies are not neutral, nor value free. Technologies, how they are configured and used, reflect the interests and values of their developers and owners. Over time, other stakeholders, including users, may become developers too by creating new applications for the technology or by adapting the technology for uses unforeseen when the technology was originally developed. An ethical impact assessment is also needed because ethical considerations are often context-dependent. What may be ethically acceptable in one context may not be acceptable in another context“ ( Wright, 2011)

This report describes how the Code of Conduct was developed, together with the complementary Ethical Impact Assessment (EIA) and Compliance Checklist. These tools operationalise the Code.

### Aim

The XR4Human project aims to co-create living guidance documents on ethical and related policy, regulatory, governance and interoperability issues of XR technologies in Europe.

### Approach

**Figure 1.0.**
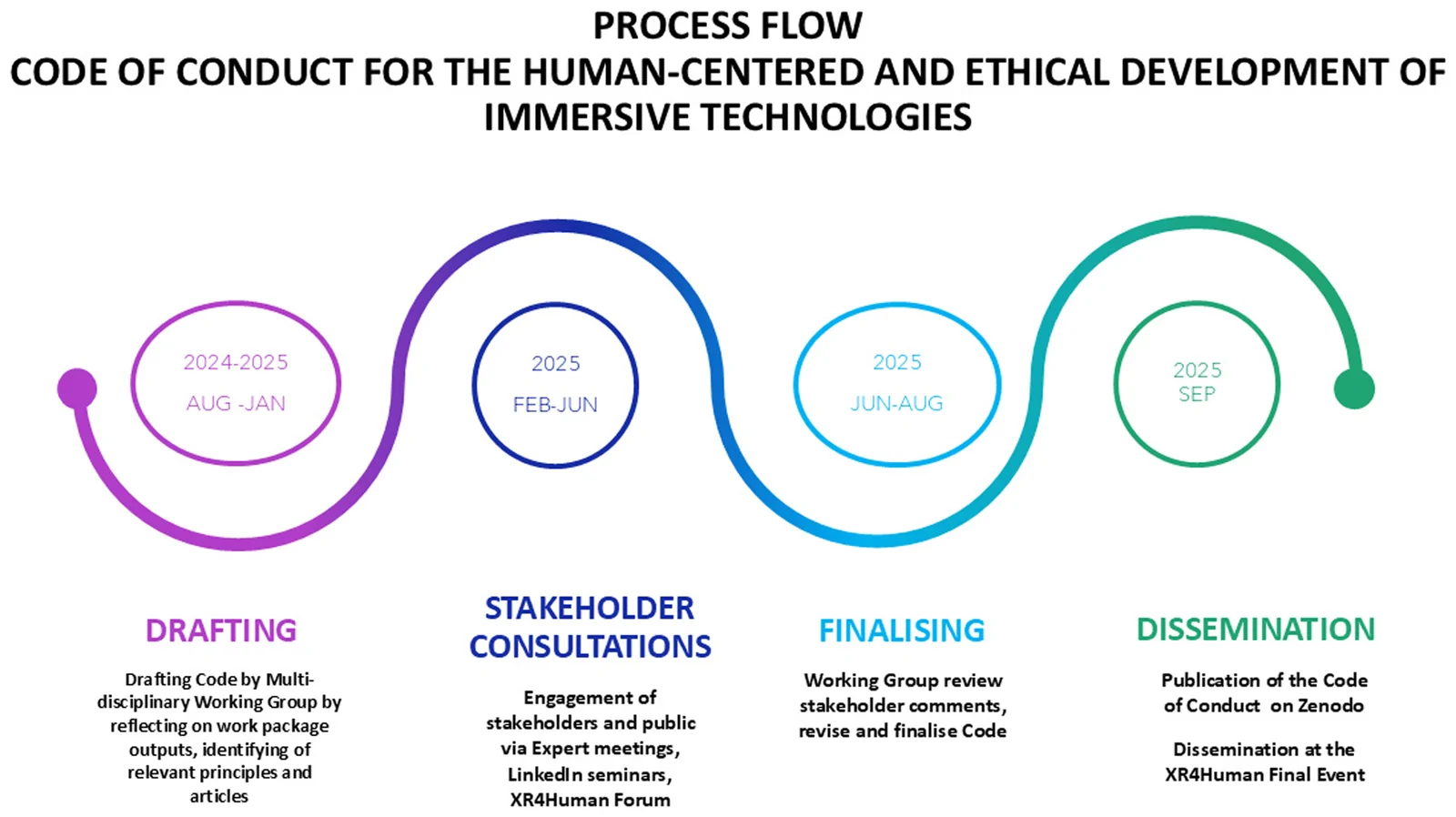
Process timeline for development of the XR4Human Code of Conduct.

The XR4Human Code of Conduct was developed using a participatory co-design methodology facilitated through expert-led workshops as well as stakeholder and public consultations ( Brouwers *et al.*, 2010). A multidisciplinary working group of WP leaders and researchers drafted, refined and finalised the Code of Conduct through a series of in person/virtual workshops held over a 12 month period ( Figure 1.0). The initial brainstorming and drafting by experts took place in August 2024 and continued until January 2025, Stakeholder consultations were conducted February to May 2025 and final editing and publication between June and August 2025. The process entailed reflecting on the recommendations of the individual WP deliverables, then identifying and drafting of guiding principles and corresponding articles. The draft was then circulated for consultation with stakeholders (policy makers, developers, users) via email, targeted expert Zoom meetings, the XR4Human forum, and on Linkedin. Stakeholder consultations were facilitated by XR4Europe and the Communication and Dissemination lead with support from researchers from USN and UiO. Sample prompts were used in the XR4Human forum and LinkedIn to stimulate engagement on clarity, practicality, and comprehensiveness of the Code ( Table 1). All feedback, including those from members of the consortium, was logged and reviewed weekly. Proposed changes were tracked, discussed, and informed iterative revisions of the draft. Additional meetings were held to consider the cumulative comments-first to finalise the principles, then the articles. The working group was divided into smaller subgroups; each assigned a specific area to assess potential revisions. Each subgroup presented its recommendations to the full working group and consensus was reached on the final content.

**Table 1.** Sample prompts used in the XR4Human forum and LinkedIn to stimulate engagement on clarity, practicality, and comprehensiveness of the Code.

1. Are there any sections of the Code of Conduct that are unclear or need further explanation? 2. Is the language used in the Code of Conduct clear, concise, and easily understandable for a broad audience, including developers, policymakers, and end-users? 3. Are there any terms or concepts in the Code of Conduct that require additional definitions or clarification? 4. Are any sections overly technical or complex for the intended audience? 5. How practical do you find the guidelines in the Code of Conduct for real-world application? 6. What potential challenges do you foresee in implementing this Code of Conduct in real-world scenarios? How can these challenges be addressed? 7. Do you see any potential conflicts between the Code’s principles and current business models or industry practices? 8. What compliance mechanisms or enforcement strategies would help ensure effective implementation of the Code?

## Synthesis of the Ethical Impact Assessment (EIA) and compliance checklist

Concurrent with the process of developing the CoC, a subgroup was formed to draft the Ethical Impact Assessment (EIA) and a compliance checklist. To achieve this, the principles and corresponding articles of the draft CoC were organized under five areas of impact for comprehensiveness and ease of understanding. The deliverables from the XR4human project were uploaded to Google Notebook LLM ( Google (n.d.)) and prompts used to generate questions for ethically relevant risks under each area of impact and the corresponding principles and articles noted. This ensured that the questions for the EIA were derived exclusively based on the outputs of the XR4Human project. Weekly meetings were held to ensure the questions under each area of impact were clear and relevant. Any overlaps or ambiguities identified were discussed, refined or removed based on consensus. Once there were no further disagreements, the document was finalized, and the compliance checklist was created. The EIA and checklist were shared with the members of the consortium via Teams for feedback, refinement and finalisation. Finally, the compliance checklist was designed as a one-page document that could be used as a quick reference point of reflection.

### Principles

The following principles of the Code of Conduct are reported under nine domains:

1. Human-centred design
2. Diversity, inclusivity, accessibility, and equitability
3. Trustworthiness and transparency
4. Privacy and data governance
5. Safe experience
6. Identity and right to anonymity
7. Sustainability
8. Technical security and interoperability
9. Moral-dilemma resolution and Risk management.

### Articles

 Seven articles set out how the above principles are to be applied in practice:

1. User transparency by design
2. Data protection and privacy by design
3. Risk management by design
4. Well-being by design
5. Identity, personas, and avatars
6. Shared spaces
7. Engagement with non-human resources.

### Five key areas of impact

The accompanying EIA for immersive technologies and the compliance checklist focus on five key areas:

1. Health and well-being
2. Autonomy
3. Data and technology
4. Social
5. Environmental

Together, the nine principle domains, seven articles, and five areas of impact provide a structured basis for designing, assessing, and iteratively improving XR technology products and experiences.

### Operationalisation and dissemination

**Figure 2.0.**
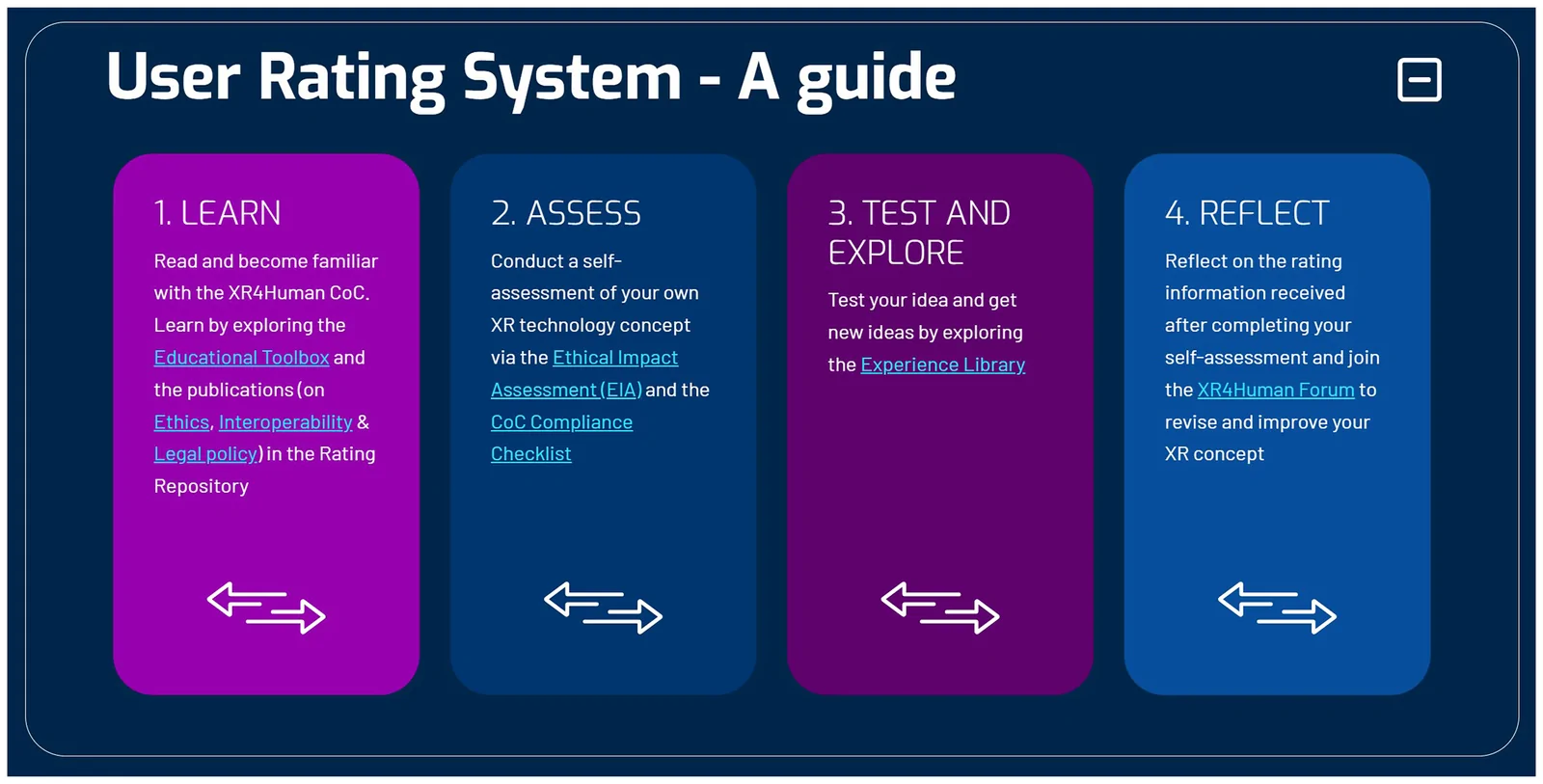
A stepwise approach to operationalise the XR4Human Code of Conduct.

An infographic was developed by applying learning by doing educational pedagogy ( Morris, 2020). The infographic provides a stepwise approach to the developer/user to 1) Learn, 2) Assess, 3) Test and Explore, and 4) Reflect ( Figure 2.0). Developers and users are encouraged to familiarise themselves with the project’s educational outputs as well as the CoC, then conduct a self-assessment of their XR technology idea, product, or experience using the EIA and compliance checklist as part of the Rating Repository. Where a product is in development, it can be tested and explored via submission to the XR4Human Experience library with optional engagement through the XR4Human forum. Insights from testing inform reflection and revision as deemed necessary by the developer. This iterative cycle operationalises the CoC and EIA and aligns with the reflective equilibrium approach in ethics ( Knight, 2025).

## Ethics and consent

Ethics and consent were not required as this is a paper proposing guidelines for developers and users of XR and as such did not involve research participants and personal identifiable data.

## Data Availability

This article does not involve the collection, generation, or analysis of empirical data. It presents a code of conduct based on ethical analysis, existing standards, and professional norms. Data are available under the terms of the Creative Commons Attribution 4.0 International license (CC-BY 4.0).  The Code of Conduct, Ethical Impact Assessment and Compliance Checklist are available in the Zenodo repository: Bernabe, R., Baraas, R., Barngrover, M., Cox, S., Hagen, L. A., Kadlubsky, A., Kavouras, P., Krastina, I., Ladikas, M., Amoros Lark, M., Correa Pérez, M., Stephane, L., & Schreer, O. (2025). XR4HUMAN code of conduct for the human-centered and ethical development of immersive technologies (Version v1) [Standard]. Zenodo. https://doi.org/10.5281/zenodo.17035113
