# From principles to practice: A code of conduct for the human-centered and ethical development of immersive technologies

**DOI:** 10.12688/openreseurope.22230.2

**Published:** 2026-07-14

**Authors:** XR4HUMAN (The Equitable, Inclusive, and Human-Centered XR), Rosemarie De La Cruz Bernabe, Rigmor C. Baraas, Shereen Cox, Lene A. Hagen

**Affiliations:** 1 Centre for Medical Ethics, Institute of Health and Society, Faculty of Medicine, University of Oslo, Oslo, Norway; 2 National Centre for Optics, Vision and Eye Care, University of South-Eastern Norway, Kongsberg, Buskerud, Norway

**Keywords:** eXtended reality, Immersive technologies, Ethics, Virtual reality, Virtual worlds, Code of Conduct, Augmented reality, Mixed reality

## Abstract

The XR4Human Code of Conduct was developed over a 12-month period using a participatory co-design approach that combined multi-disciplinary expert workshops with members of the consortium followed by stakeholder and public consultations. The process was intentionally iterative. Drafting, review, and revision were guided by reflective equilibrium to reconcile empirical inputs from the project deliverables, normative principles, and practical constraints. The final Code is organised into nine domains that consolidate core principles relevant to the responsible design, development, and deployment of XR systems. These principles are made more concrete through seven articles that specify actionable obligations. To support real-world adoption, the Code is operationalised via an Ethical Impact Assessment and a companion Compliance Checklist aligned to the core principles and articles.

## Disclaimer

The views expressed in this article are those of the authors. Publication in Open Research Europe does not imply endorsement of the European Commission.

## Introduction

Immersive technologies aka eXtended reality (XR) is fast becoming a highly relevant technology with a wide range of applications spanning different industries. XR includes Augmented Reality (AR), Mixed Reality (MR), and Virtual reality (VR) where AR is a digital overlay in physical spaces and is the least immersive experience, MR enables interactions with digital elements in physical spaces, and VR is completely immersive experience ( Milgram & Kishno, 1994). XR technologies span various industries and use cases such as entertainment including gaming, research, education and training in military and healthcare industries, digital commerce, socialising and tourism ( Voinov *et al.*, 2024). Concurrent with the development and the applications are various ethical and legal challenges. The Equitable, Inclusive, and Human-Centered XR project ( XR4Human) had a goal to map various legal, ethical and interoperability challenges of XR technologies and propose guidance for developers and users of XR embedded in human centred and ethics by design principles. To achieve this, researchers in the project employed within seven work packages (WP) over a period of three years deep dived into XR literature and conducted stakeholder surveys and interviews to clearly elucidate and articulate ethical, legal and technological issues. These were published on the XR4human website and in peer reviewed journals. Reports include mapping of a) ethical issues in XR (WP 2), b) regulatory and governance issues (WP 3), c) existing and emerging interoperability and seamless integration of XR (WP4), and d) challenges and presentation of ethical, legal regulatory and technical interoperability solutions ( XR4HUMAN, n.d.). Ethical and legal issues of XR were mapped across several work packages. Of significance are the issues associated with constant data capture, invasion of privacy, and risk of manipulation due to the inherent features of XR output devices ( Cox *et al.*, 2025). Human rights issues, such as inclusivity and accessibility, may also arise with XR if a subset of the population is not culturally represented or is unable to access or use the devices because of age or varying types of physical and mental disabilities ( Cox *et al.*, 2025). There are also societal, environmental and economic concerns such as increased risk of crime and anti-social behaviours such as stalking or virtual assaults, cybercrimes or real-world implications arising from virtual social interactions ( Cox *et al.*, 2025). Economic disparity exists also where some groups in society will advance with the use of XR technology while others would be disadvantaged due to financial challenges ( Cox *et al.*, 2025). WP2 also mapped and reported risks and harms associated with XR technology of ethical relevance using a risk typology under five themes: Health and Well-Being, Autonomy, Epistemic, Normative, Structural, and Technical Safety and Security. Health and well-being risks include cognitive, physical, psycho-emotional, and socio-relational harms. Some XR users may experience cybersickness, injuries secondary to falls, experience challenges with entering and exiting virtual and real spaces. False memories may be created where users are unable to distinguish between what they experience in the virtual space from what actually occurred in the physical world. Autonomy risks include issues of privacy, self-sovereignty, surveillance, and authenticity. Some XR experiences have the ability to capture eye movements, lip smacking, and other micro emotions unknown to the user. Epistemic risks of XR include issues of uncertainty and risk of manipulation. Normative risks include norm and rights violations while structural and technical safety/security risks include legal, socio-political, economic/financial and risks of hacking or dual use of technology ( Voinov *et al.*, 2024). Legislation and interoperability standards for XR technologies are also lagging. What obtains are Codes of Conduct or guidance tools proposed mainly by academics or industry stakeholders ( Cox *et al.*, 2025). The combined recommendations of these reports needed to be translated into a normative framework that could be used by XR industry stakeholders from idea to development and deployment. To this end, the normative output of the project is a Code of Conduct (CoC) for human-centered and ethical development of immersive technologies ( Bernabe *et al.*, 2025).

Additionally, it is important that the CoC is not a document that provides superficial guidance but one which is practical for seamless adoption by developers and users. This approach aligns with translational ethics which bridges theoretical, empirical and practical aspects of ethics i.e. ensuring the actual application of ethical principles to real world situations ( Bærøe, 2014). One way to bridge the gap between theory and practice is the use of impact assessments. Impact assessments have become commonplace for effecting standards in various industries including artificial intelligence, risk management and privacy ( Trilateral Research, 2020; Wernick, 2024). The approach is to consider relevant principles, values or issues of an area of impact drawing from various sources, then create questions that would cause one to reflect on the impact of the idea/work in progress ( Wright, 2011). In the context of ethical impact assessment of technology, David Wright notes:

“an ethical impact assessment is needed of new and emerging technologies because technologies are not neutral, nor value free. Technologies, how they are configured and used, reflect the interests and values of their developers and owners. Over time, other stakeholders, including users, may become developers too by creating new applications for the technology or by adapting the technology for uses unforeseen when the technology was originally developed. An ethical impact assessment is also needed because ethical considerations are often context-dependent. What may be ethically acceptable in one context may not be acceptable in another context” ( Wright, 2011).

This open letter describes how the Code of Conduct was developed, together with the complementary Ethical Impact Assessment (EIA) and Compliance Checklist. These tools operationalise the Code.

### Aim

The XR4Human project aims to co-create living guidance documents on ethical and related policy, regulatory, governance and interoperability issues of XR technologies in Europe.

### Approach

**Figure 1.**
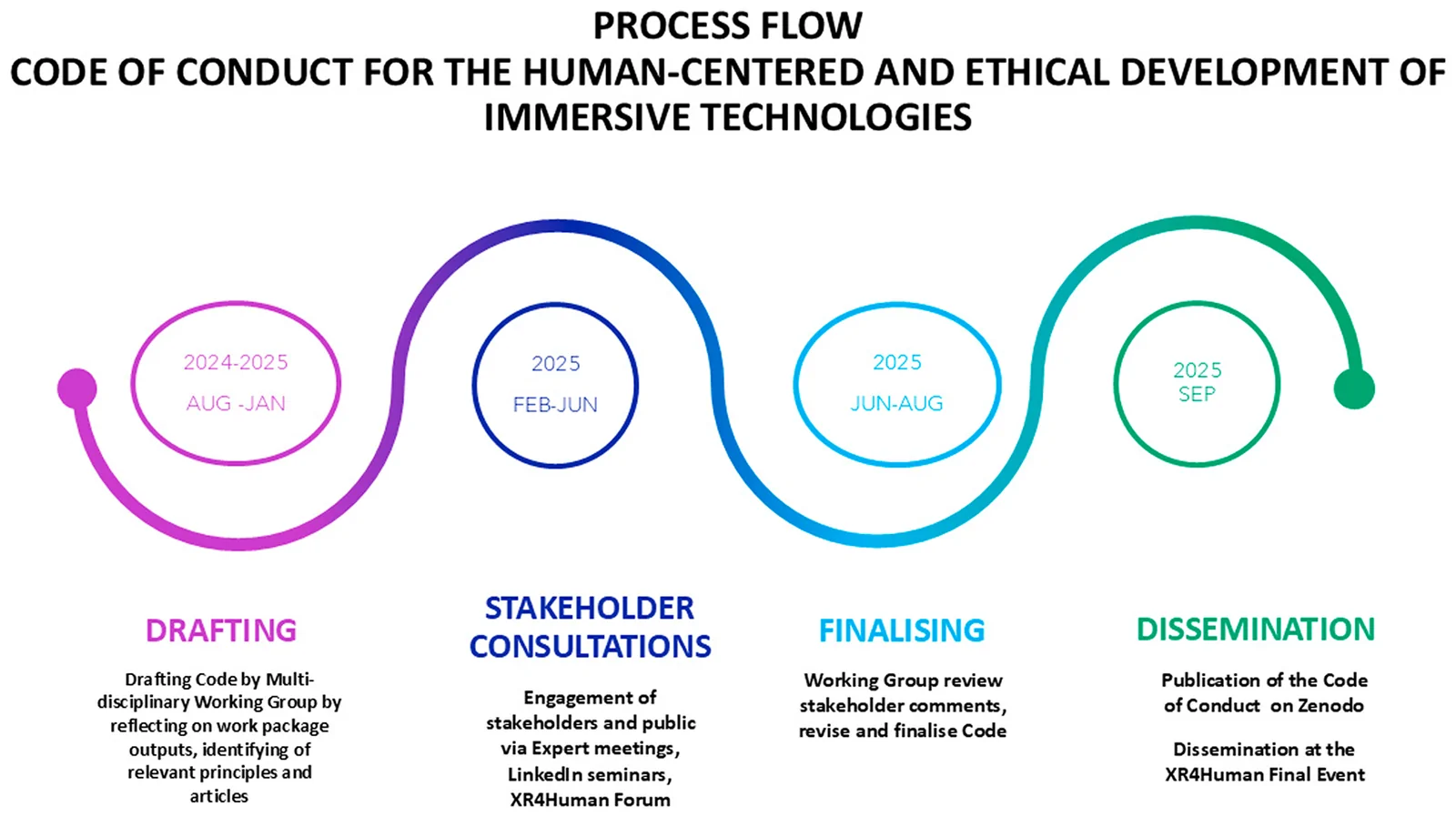
Process timeline for development of the XR4Human Code of Conduct.

The XR4Human Code of Conduct was developed using a participatory co-design methodology facilitated through expert-led workshops as well as stakeholder and public consultations ( Brouwers *et al.*, 2010). A multidisciplinary working group of WP leaders and researchers (n = 18) drafted, refined and finalised the Code of Conduct through a series of in person/virtual workshops held over a 12 month period ( Figure 1). The initial brainstorming and drafting by experts took place in August 2024 and continued until January 2025. The working group included XR technology experts, vision scientists, ethicists, philosophers, lawyers, and researchers. Stakeholder consultations were conducted from February to May 2025, followed by editing from June until publication in August 2025. The process entailed reflecting on the recommendations of the individual WP deliverables, then identifying and drafting of guiding principles and corresponding articles. The draft was then circulated for consultation with stakeholders (policy makers, developers, users) via email, targeted expert Zoom meetings, the XR4Human forum, and on Linkedin. In addition, stakeholder engagement were facilitated by XR4Europe and the Communication and Dissemination WP lead with support from researchers from the University of South-East Norway (USN), OpenAR Cloud, Europe, and the University of Oslo, Norway (UiO). Three online workshops were held on 4, 6 and 13 May 2025. Stakeholders were invited via a Google Forms survey. The workshop attendees included representatives from the training and education sector; software development (software as a service/SaS); industry associations (France Immersive Learning, for example); researchers in computer science, engineering, design and psychology. Nationalities represented were French, Norwegian, German, Italian, Spanish, British, Irish, Dutch, Belgian, and Greek. The online seminars discussing the CoC were hosted by XR4Europe. During these seminars, researchers were asked for feedback, and suggestions were collected on how to improve the CoC.

Code of Conduct Promotion and Stakeholder Consultation via LinkedIn:

• *Guidance or Governance?* – Apr 29, 2025–168 Unique Views – 256 Minutes Viewed - https://www.linkedin.com/events/7313524939150491648/
• *The Language of Ethics* – Apr 22, 2025–237 Unique Views – 617 Minutes Viewed - https://www.linkedin.com/events/7313521359827947520/
• *What Guides Ethical XR?* – Apr 15, 2025–279 Unique Views – 870 Minutes Viewed - https://www.linkedin.com/events/7313517658300166144/

Sample prompts were used in the XR4Human forum and LinkedIn to stimulate engagement on clarity, practicality, and comprehensiveness of the Code ( Table 1). Stakeholders had the opportunity to comment directly on the CoC draft using the document’s commenting feature. One researcher compiled the comments into a single report. All feedback, including those from members of the consortium, was logged and reviewed weekly. Proposed changes were tracked, discussed, and informed iterative revisions of the draft. Additional meetings were held to consider the cumulative comments-first to finalise the principles, then the articles. The working group was divided into smaller subgroups; each assigned a specific area to assess potential revisions. Each subgroup considered the inputs received from stakeholders then presented its recommendations to the full working group where consensus was reached on the final content. Comments that were not incorporated were either already addressed in some form in the Code or were outside its scope (for example, issues of data protection covered under General Data Protection Regulations, GDPR).

**Table 1.** Sample prompts used in the XR4Human forum and LinkedIn to stimulate engagement on clarity, practicality, and comprehensiveness of the Code.

1. Are there any sections of the Code of Conduct that are unclear or need further explanation? 2. Is the language used in the Code of Conduct clear, concise, and easily understandable for a broad audience, including developers, policymakers, and end-users? 3. Are there any terms or concepts in the Code of Conduct that require additional definitions or clarification? 4. Are any sections overly technical or complex for the intended audience? 5. How practical do you find the guidelines in the Code of Conduct for real-world application? 6. What potential challenges do you foresee in implementing this Code of Conduct in real-world scenarios? How can these challenges be addressed? 7. Do you see any potential conflicts between the Code’s principles and current business models or industry practices? 8. What compliance mechanisms or enforcement strategies would help ensure effective implementation of the Code?

### Normative analysis and synthesis of principles

Earlier, a description of the methodological approach resulting in the XR4Human CoC was outlined. This section examines in greater depth the normative reflection and synthesis that resulted in the identification of five areas of impact, nine principles, and seven practical articles (see Table 2). The project’s collective outputs concerning the impact of XR technologies are grouped into five domains: health and well-being, autonomy, data and technology, social, and environmental. These domains were derived from an analysis of the empirical findings generated throughout the project, particularly the mapping of ethical and legal issues and the identification of risks associated with the development and use of XR, and how these may affect individuals and society. After identifying the ethically significant challenges of XR in relation to these domains, members of the consortium explored how these could be addressed through ethical principles. Through an iterative process of discussion, comparison, and revision, the consortium sought to achieve coherence between empirical evidence and the moral judgements of the experts and stakeholders. This adapted reflective equilibrium approach facilitated the identification of relevant principles and accompanying articles ( Knight, 2025), which formed the basis of the first draft of the CoC. Stakeholder feedback was then solicited, followed by additional discussions on how to incorporate stakeholders’ perspectives, then finally the refinement completion of the Code of Conduct. In the following section, we briefly explain how this approach was applied to each domain.

**Table 2.** XR4Human Code of Conduct’s areas of impact, principles and corresponding articles.

Areas of impact	Principles	Articles
1. Health and well-being 2. Autonomy 3. Data and technology 4. Social 5. Environmental	1. Human-centred design 2. Diversity,inclusivity, accessibility, and equitability 3. Trustworthiness and transparency 4. Privacy and data governance 5. Safe experience 6. Identity and right to anonymity 7. Sustainability 8. Technical security and interoperability 9. Moral-dilemma resolution and Risk management	1. User transparency by design 2. Data protection and privacy by design 3. Risk management by design 4. Well-being by design 5. Identity, personas, and avatars 6. Shared spaces 7. Engagement with non-human resources

The first domain of significance was health and well-being. Given that the use of XR devices, or immersion in XR experiences, can lead to physical and psychological harm, health and wellbeing was considered a highly significant impact domain. Adopting the reflective framework, the ethical principles of non-maleficence (minimising harm) and beneficence (promoting well-being) were considered particularly relevant ( Voinov *et al.*, 2024). Participants emphasized that developers of XR technologies should not only focus on the intended purpose of XR technology and its applications but also consider how best to minimise negative impacts on health and, where possible, promote well-being ( Voinov *et al.*, 2024). During the discussions, it was noted that developers are not ethicists, and terminology should align with their context. Consequently, human-centred design was identified as a pertinent approach to mitigating potential physical, cognitive, and psychological risks. Human-centred design, a concept from the field of technology and engineering, is defined as “an approach to interactive systems development that aims to make systems usable and useful by focusing on the users, their needs and requirements, and by applying human factors/ergonomics, and usability knowledge and techniques” ( ISO, 2019). This approach enhances effectiveness and efficiency, improves human well-being, user satisfaction, accessibility and sustainability; and counteracts possible adverse effects of use on human health, safety and performance ( ISO, 2019; Bernabe *et al.*, 2025).

The goal is not merely to avoid harm but to guide developers to critically reflect on the design features of XR output devices and experiences, and to implement measures that proactively promote human well-being while deployed. In practice, a human-centred design means prioritising ergonomics, usability, accessibility, and safe immersion. The specific requirements for operationalising the principle of human centred design in XR are outlined in the accompanying article well-being by design ( Bernabe *et al.*, 2025).

The second area of impact focuses on autonomy, with particular attention to issues of identity and control. Identity relates to the ability of users to create and maintain a unique expression of self in an XR environment, especially in shared spaces where their identity is personified through avatars. Autonomy in relation to control refers to the user’s ability to make decisions without manipulation and of their own volition ( Voinov *et al.*, 2024). This is also relevant to bystanders within an XR environment or experience ( O’Hagan *et al.*, 2023). Reflecting on the issues of autonomy, the consortium adopted the principles of identity and right to anonymity, as well as trustworthiness and transparency. Together, these principles were considered important to support XR users’ ability to exercise meaningful control over their digital representations and experiences within XR spaces ( Bernabe *et al.*, 2025).

The third area of impact is data and technology. This domain concerns the type and volume of data that can be extracted through XR output devices, and how this data is managed, stored, and potentially shared with third parties. XR devices rely on the collecting of highly sensitive information, including biometric and behavioural data. Consideration must therefore extend not only to the data of immediate users, but also to that of bystanders, whose data may be knowingly or unknowingly extracted with or without their consent. The normative principles applied to this domain are privacy by design and data governance ( Bernabe *et al.*, 2025). It is well-established that XR technologies have the capability to extract substantial amounts of data through various output devices. Although many jurisdictions, especially in the European Union, are governed by strict data protection laws, ethical safeguards remain crucial for protecting the rights of users and bystanders, especially to prevent violation of trust ( Voinov *et al.*, 2024; Cox *et al.*, 2025).

The remaining two areas of impact are social and environmental. The social impact of XR was considered highly significant, as the economic and didactic benefits are often emphasized while the challenges such as discriminating against persons of varying abilities and ethnicities, as well as economic disparities commonly referred to as a digital or technology divide tend to be underreported. To address these issues, the consortium adopted the principles of diversity, inclusivity, accessibility, and equitability ( Bernabe *et al.*, 2025). Another important consideration is that of shared XR spaces, such as virtual worlds, often referred to as the metaverse. These interconnected spaces, in which users interact through avatars to learn, socialise and collaborate, can be a double-edged sword: they offer substantial benefits but also entail risks, including harassment and bullying. After careful contemplation of the benefits and risks, the consortium identified the need for a safe experience in such shared spaces, to foster a respectful environment which minimises the potential for psychosocial harm ( Bernabe *et al.*, 2025). Finally, the environmental impact of XR technologies encompasses the production, use, and disposal of devices. Accordingly, sustainability was adopted as the guiding principle, with an aim of reducing or mitigating, as far as reasonably possible, any long-term environmental damage associated with XR technologies ( Bernabe *et al.*, 2025).

## Synthesis of the Ethical Impact Assessment (EIA) and compliance checklist

Concurrent with the process of developing the CoC, a subgroup was formed to draft the Ethical Impact Assessment (EIA) and a compliance checklist. To achieve this, the principles and corresponding articles of the draft CoC were organized into five areas of impact to enhance comprehensiveness and ease of understanding. The deliverables from the XR4Human project were uploaded to Google Notebook LM (Model-Gemini 2.5 Flash) and a master prompt used to generate questions for ethically relevant risks under each area of impact, with the associated principles and articles noted. This ensured that the questions for the EIA were derived exclusively based on the outputs of the XR4Human project. The master prompt instructed the LM to generate impact descriptors with relevant questions for developers and end-users with emphasis on potential effects, risks, and harms of XR technologies. For each impact category, the questions were required to address risks, harms, safeguards, and individual user considerations, thereby promoting developer and user awareness and informing decision making. Following this initial use of the LM to generate a draft, weekly meetings were held to review the questions under each area of impact to ensure clarity and relevance. Any overlaps or ambiguities were discussed, then refined or removed based on consensus among the researchers. Once no further disagreements remained, the document was finalized, and the compliance checklist was created. The Google Notebook LM was used solely for initial generation of questions based on areas of impact, accompanying principles and related articles to ensure the questions were originating from the project deliverables. Google LM was selected because it is a more focused AI research tool and therefore considered less likely to produce hallucinations than general-purpose LLMs. Additionally, a human oversight approach was implemented through a multi-researcher review and refinement procedure. The EIA and checklist were subsequently shared with the members of the consortium via Microsoft Teams for feedback, further refinement and finalisation. Finally, the compliance checklist was designed as a one-page document that could be used as a quick reference tool for reflection.

In summary, the consortium’s judgement supported by the feedback from key stakeholders, is that the XR4Human Code of Conduct’s five areas of impact, nine principle domains and, seven corresponding articles provide a structured basis for designing, assessing, and iteratively improving XR technology products and experiences.

### Operationalisation and dissemination

**Figure 2.**
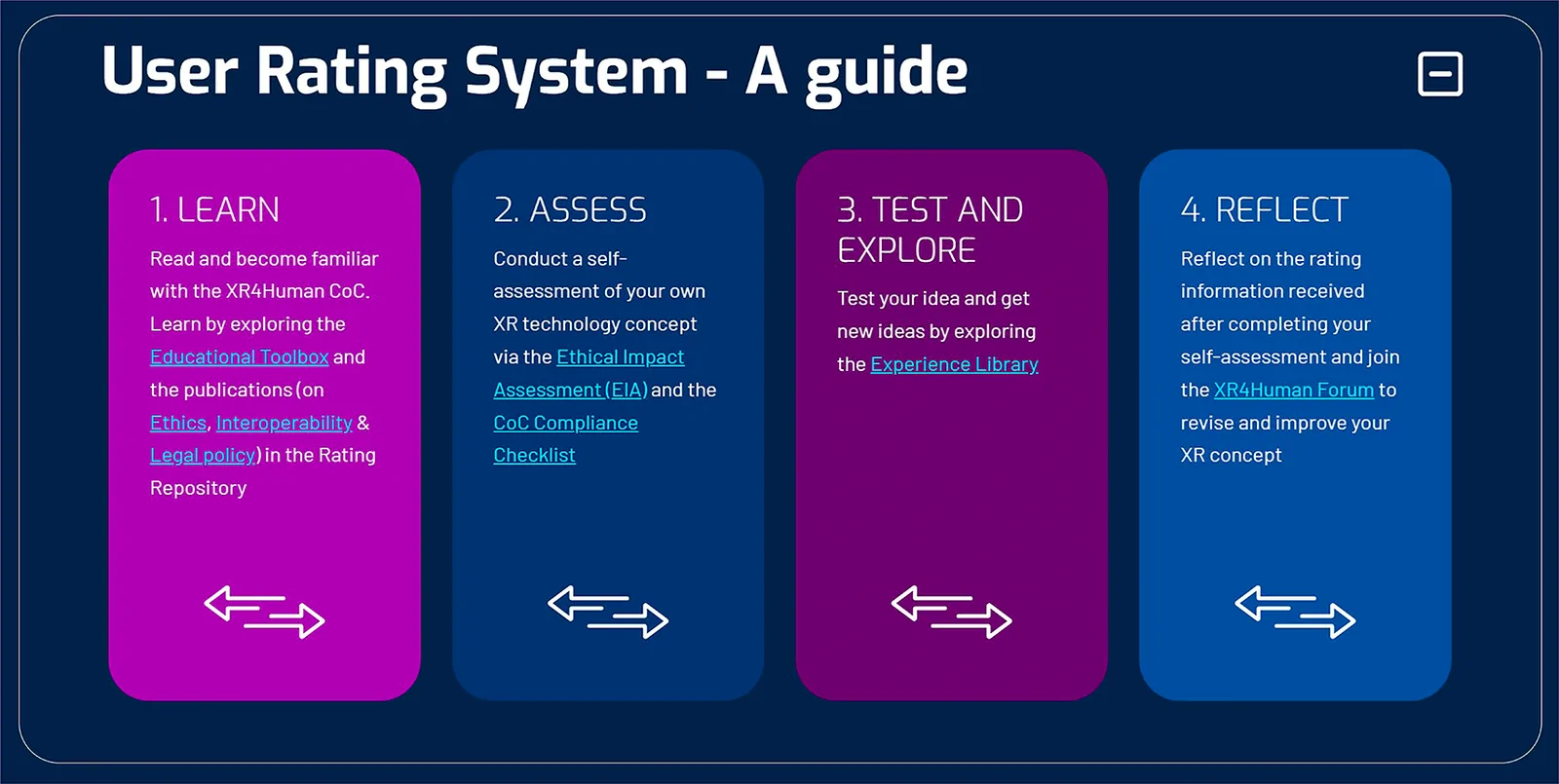
A stepwise approach to operationalise the XR4Human Code of Conduct.

An infographic was developed by applying learning by doing educational pedagogy (Morris, 2020). The infographic provides a stepwise approach to the developer/user to 1) Learn, 2) Assess, 3) Test and Explore, and 4) Reflect ( Figure 2). Developers and users are encouraged to familiarise themselves with the project’s educational outputs as well as the CoC, then conduct a self-assessment of their XR technology idea, product, or experience using the EIA and compliance checklist as part of the Rating Repository. The CoC may be applicable to healthcare, education and training, gaming/entertainment and ecommerce use cases. Where a product is in development, it can be tested and explored via submission to the XR4Human Experience library with optional engagement through the XR4Human forum. Insights from testing inform reflection and revision as deemed necessary by the developer. This iterative cycle operationalises the CoC and EIA and aligns with the reflective equilibrium approach ( Knight, 2025).

## Ethics and consent

Ethics and consent were not required as this is a paper proposing guidelines for developers and users of XR and as such did not involve research participants and personal identifiable data.

## Contributors

Leon Kester, Faisal Mushtaq, Franziska Sörgel, Cristiana-Anca Voinov, Eleni Spyrakou.

## Data Availability

This article does not involve the collection, generation, or analysis of empirical data. It presents a code of conduct based on ethical analysis, existing standards, and professional norms. Data is available under the terms of the Creative Commons Attribution 4.0 International license (CC-BY 4.0). The Code of Conduct, Ethical Impact Assessment and Compliance Checklist are available in the Zenodo repository: Bernabe, R., Baraas, R., Barngrover, M., Cox, S., Hagen, L. A., Kadlubsky, A., Kavouras, P., Krastina, I., Ladikas, M., Amoros Lark, M., Correa Pérez, M., Stephane, L., & Schreer, O. (2025). XR4HUMAN code of conduct for the human-centered and ethical development of immersive technologies (Version v1) [Standard]. Zenodo. https://doi.org/10.5281/zenodo.17035113.
