# Supplementary material for: From principles to practice: A code of conduct for the human-centered and ethical development of immersive technologies
Source: Open Res Eur. 2026 Jul 14;6:35. Originally published 2026 Feb 3. [Version 2] doi: 10.12688/openreseurope.22230.2 (PMC13554789; doi:10.12688/openreseurope.22230.2)
Supplement: Peer review history

# Reviewer report: Reviewer response for version 2

**DOI:** 10.21956/openreseurope.26485.r77655

**Published:** 2026-08-06

**Authors:** Siti Sahronih

**Affiliations:** 1 Institut Prima Bangsa, Cirebon, Indonesia

The authors of this open letter present a research contribution related to the values and principles underlying the development of XR technology, resulting in a Code of Conduct, an Ethical Impact Assessment (EIA), and a Compliance Checklist designed for practical application. The development process, which utilized participatory co-design and involved experts from various multidisciplinary fields as well as appropriate consultation with stakeholders, adds significant value to the research. 

 The introduction to this research paper also provides a solid background on XR. It identifies various risks, such as privacy, manipulation, health, inclusion, security, and social impact. Additionally, it connects these issues to the deliverables of the XR4Human project.

 However, there are several areas the authors need to improve regarding a brief comparison of the proposed framework with other XR related research frameworks. This would help clarify the research gaps and ensure this article serves as a well positioned contribution to the literature for future researchers. This is because there is already a significant body of research on XR ethics frameworks, IEEE, XRSI, EU AI governance, and so on. The authors also merely list **codes of conduct** **exist **without providing any practical analysis. Adding brief discussions of practical examples regarding XR applications evaluated using the EIA and Compliance Checklist would enhance clarity, reproducibility, and ease of use.

 Thus, overall, the following points could be revised: 

 (1) there is a need for more in depth research gap on existing ethical and governance frameworks for XR; (2) provide a more detailed explanation of stakeholder participation and the consensus building process; (3) include concrete examples demonstrating how conflicting ethical considerations are resolved using the reflective equilibrium approach; (4) add a brief description of examples illustrating the application of an Ethical Impact Assessment (EIA) and a Compliance Checklist in representative XR use cases.

Where applicable, are recommendations and next steps explained clearly for others to follow? (Please consider whether others in the research community would be able to implement guidelines or recommendations and/or constructively engage in the debate)

Yes

Does the article adequately reference differing views and opinions?

Yes

Are all factual statements correct, and are statements and arguments made adequately supported by citations?

Yes

Is the rationale for the Open Letter provided in sufficient detail? (Please consider whether existing challenges in the field are outlined clearly and whether the purpose of the letter is explained)

Partly

Is the Open Letter written in accessible language? (Please consider whether all subject-specific terms, concepts and abbreviations are explained)

Yes

Reviewer Expertise:

Augmented reality, immersive technologies, mixed reality, virtual reality.

I confirm that I have read this submission and believe that I have an appropriate level of expertise to confirm that it is of an acceptable scientific standard, however I have significant reservations, as outlined above.

# Reviewer report: Reviewer response for version 2

**DOI:** 10.21956/openreseurope.26485.r77424

**Published:** 2026-08-06

**Authors:** Catherine Flick

**Affiliations:** 1 Staffordshire University, Stoke-on-Trent, England, UK

I thank the authors for their significant updates - I am now more satisfied with the purpose of this article and understand the underlying work that has gone into it. 

 I remain significantly concerned about the lack of participant consent in the development of this work, however - you say you requested feedback from LinkedIn and your forum outside of your existing team. While I appreciate that the Norwegian Health Research Act has no specific requirements for this kind of activity for health-related research, I would argue that this project would not fall within that scope as it is not health-related. Additionally, there are several existing standards across the research space (especially in human-centred technology research) that would require such consent procedures to take place, many of which are listed here: https://research-and-innovation.ec.europa.eu/strategy/support-policy-making/scientific-support-eu-policies/research-ethics-and-integrity_en

 I really hesitate to change my review on this as it feels very problematic for the development of a code of conduct/ethics to not hold itself to the highest of ethical procedures itself. We cannot pick and choose the parts that are most amenable to us when we develop these approaches lest we be seen as hypocritical. 

 More detail and a reflection on the processes could help to mitigate this; as it is, it is done and perhaps some lessons could be learned from this.

Where applicable, are recommendations and next steps explained clearly for others to follow? (Please consider whether others in the research community would be able to implement guidelines or recommendations and/or constructively engage in the debate)

No

Does the article adequately reference differing views and opinions?

Partly

Are all factual statements correct, and are statements and arguments made adequately supported by citations?

Yes

Is the rationale for the Open Letter provided in sufficient detail? (Please consider whether existing challenges in the field are outlined clearly and whether the purpose of the letter is explained)

Yes

Is the Open Letter written in accessible language? (Please consider whether all subject-specific terms, concepts and abbreviations are explained)

Yes

Reviewer Expertise:

I am an ethicist of emerging technologies, currently specialising in games technologies with experience having helped to rewrite the ACM's Code of Ethics in 2018. I have also contributed to the construction of several Codes of Ethics and Codes of Conduct for smaller projects and events. I serve as Vice-Chair of the ACM Committee on Professional Ethics.

I confirm that I have read this submission and believe that I have an appropriate level of expertise to confirm that it is of an acceptable scientific standard, however I have significant reservations, as outlined above.

# Reviewer report: Reviewer response for version 2

**DOI:** 10.21956/openreseurope.26485.r77649

**Published:** 2026-08-03

**Authors:** Moses Adeleke ADEOYE

**Affiliations:** 1 Al-Hikmah University, Ilorin, Nigeria

This Open Letter describes the development of the XR4Human Code of Conduct for the human-centered and ethical development of immersive technologies, together with its associated Ethical Impact Assessment (EIA) and Compliance Checklist. The manuscript explains the participatory co-design process used to develop the Code, the normative reasoning that translated empirical findings into ethical principles, and the intended operationalization of the Code through practical assessment tools. Compared with the concerns raised during the first round of peer review, this revised version substantially strengthens the methodological description, clarifies the rationale for the work, and provides greater transparency regarding stakeholder engagement and the development process. Overall, this is a timely and valuable contribution that bridges ethical theory and practical guidance for XR developers. The manuscript is scientifically sound, although several areas could be further strengthened to improve transparency and scholarly value.

 The revised manuscript clearly explains both the challenges motivating the work and the purpose of the Open Letter. The introduction outlines major ethical, legal, social, accessibility, privacy, interoperability, and governance challenges associated with XR technologies before explaining why these findings needed to be translated into a practical Code of Conduct. The manuscript also explicitly states that the purpose of the Open Letter is to describe the development of the Code, the Ethical Impact Assessment, and the Compliance Checklist rather than merely presenting the final guidance. However, the discussion primarily presents a consensus-oriented perspective rather than engaging with alternative viewpoints or competing ethical approaches. For example: competing governance models (voluntary codes versus regulatory approaches); differing ethical frameworks that may prioritize autonomy, justice, innovation, or precaution differently; existing international XR governance initiatives from industry and standards organizations are only briefly acknowledged rather than critically compared. Including a concise discussion comparing the XR4Human approach with other existing XR ethics frameworks or standards would strengthen the manuscript and better demonstrate why this particular framework represents an advancement. This is an improvement rather than a scientific deficiency and does not undermine the manuscript's overall validity.

 One minor suggestion would be to provide additional references where statements refer broadly to current industry practice (e.g., the claim that legislation and interoperability standards are lagging) by citing more recent regulatory or standards developments. However, this is a minor enhancement rather than a substantive concern. One possible enhancement would be to include a brief worked example illustrating how a hypothetical XR application progresses through the EIA and Compliance Checklist. Such an example would improve reproducibility and facilitate uptake by practitioners.

 Points that should be addressed

 None of the following issues prevent the manuscript from being scientifically sound, but addressing them would improve its impact:

1. Include a brief comparison with other prominent XR ethics and governance frameworks to better situate the contribution within the existing landscape.
2. Expand discussion of alternative ethical perspectives or governance approaches rather than presenting only the consortium's consensus framework.
3. Include a practical worked example demonstrating application of the Ethical Impact Assessment and Compliance Checklist to a representative XR use case.
4. Where feasible, provide slightly more detail regarding how disagreements during stakeholder consultation were resolved, beyond consensus-based decision making, to further enhance methodological transparency.

Where applicable, are recommendations and next steps explained clearly for others to follow? (Please consider whether others in the research community would be able to implement guidelines or recommendations and/or constructively engage in the debate)

Yes

Does the article adequately reference differing views and opinions?

Partly

Are all factual statements correct, and are statements and arguments made adequately supported by citations?

Yes

Is the rationale for the Open Letter provided in sufficient detail? (Please consider whether existing challenges in the field are outlined clearly and whether the purpose of the letter is explained)

Yes

Is the Open Letter written in accessible language? (Please consider whether all subject-specific terms, concepts and abbreviations are explained)

Yes

Reviewer Expertise:

Educational Leadership, Research Method, ICT in Education

I confirm that I have read this submission and believe that I have an appropriate level of expertise to confirm that it is of an acceptable scientific standard, however I have significant reservations, as outlined above.

# Reviewer report: Reviewer response for version 2

**DOI:** 10.21956/openreseurope.26485.r77423

**Published:** 2026-07-30

**Authors:** Joshua A Fisher

**Affiliations:** 1 Ball State University, Muncie, Indiana, USA

I thank the authors for their revisions.

Where applicable, are recommendations and next steps explained clearly for others to follow? (Please consider whether others in the research community would be able to implement guidelines or recommendations and/or constructively engage in the debate)

Partly

Does the article adequately reference differing views and opinions?

No

Are all factual statements correct, and are statements and arguments made adequately supported by citations?

Yes

Is the rationale for the Open Letter provided in sufficient detail? (Please consider whether existing challenges in the field are outlined clearly and whether the purpose of the letter is explained)

Partly

Is the Open Letter written in accessible language? (Please consider whether all subject-specific terms, concepts and abbreviations are explained)

Yes

Reviewer Expertise:

XR UX, XR design and development, XR Interactive Storytelling, and XR Ethics

I confirm that I have read this submission and believe that I have an appropriate level of expertise to confirm that it is of an acceptable scientific standard.

# Reviewer report: Reviewer response for version 1

**DOI:** 10.21956/openreseurope.24064.r69497

**Published:** 2026-03-25

**Authors:** Catherine Flick

**Affiliations:** 1 Staffordshire University, Stoke-on-Trent, England, UK

This open letter describes the production of a code of conduct developed by the XR4Human project for extended, augmented, mixed, and virtual reality and other immersive technologies. 

 While constructing a code of conduct is a highly useful exercise it's difficult to understand what this open letter actually contributes. If it is to contribute information about the code of conduct, then it is far too brief and needs fleshing out of the Principles, Articles, and Impact sections. If it is to describe the process then it needs far more information about the process involved - how many participants, specifics of the workshops and other interactions, which experts contributed, etc. It feels like these are available elsewhere, in other papers, so it's difficult to understand the purpose of this article. 

 As it is, it is difficult to know what one actually *does* with this article, given there is no detail for what those reading it should do outside of simple headings. 

 I also find it quite surprising that the authors claim there is no need for ethics and consent when they describe a human research activity with several rounds of feedback gathered throughout the process from different stakeholders. There is also no critical assessment of the use of large language models in the work which are known for their inaccuracies. LLM use is controversial in research methods, so an uncritical use of it is worth questioning the usefulness of that over human work.

Where applicable, are recommendations and next steps explained clearly for others to follow? (Please consider whether others in the research community would be able to implement guidelines or recommendations and/or constructively engage in the debate)

No

Does the article adequately reference differing views and opinions?

Partly

Are all factual statements correct, and are statements and arguments made adequately supported by citations?

Yes

Is the rationale for the Open Letter provided in sufficient detail? (Please consider whether existing challenges in the field are outlined clearly and whether the purpose of the letter is explained)

Yes

Is the Open Letter written in accessible language? (Please consider whether all subject-specific terms, concepts and abbreviations are explained)

Yes

Reviewer Expertise:

I am an ethicist of emerging technologies, currently specialising in games technologies with experience having helped to rewrite the ACM's Code of Ethics in 2018. I have also contributed to the construction of several Codes of Ethics and Codes of Conduct for smaller projects and events. I serve as Vice-Chair of the ACM Committee on Professional Ethics.

I confirm that I have read this submission and believe that I have an appropriate level of expertise to state that I do not consider it to be of an acceptable scientific standard, for reasons outlined above.

# Response

**Published:** 2026-06-29

**Authors:** Shereen Cox

**Affiliations:** Universitetet i Oslo, Oslo, Oslo, Norway

The open letter serves as an introduction to the Code of Conduct which is linked to the letter. The intention of the letter is to give an overview of the process of development of the Code, while the intent is to draw attention to the Code of Conduct document.   With regards to ethics approval/waiver, according to Norwegian Health Research Act, stakeholder workshops are not classified as a research-based activities. This was intended to be a strictly consultative process with stakeholders. However, GDPR measures would take priority when processing personal identifiable information. The activities surrounding the Code development were mainly expert meetings and stakeholder engagement. Details for this have been added to the letter.

# Reviewer report: Reviewer response for version 1

**DOI:** 10.21956/openreseurope.24064.r69504

**Published:** 2026-03-25

**Authors:** Frank Ursin

**Affiliations:** 1 Hannover Medical School, Hanover, Germany

This Open Letter describes how the XR4Human consortium developed a Code of Conduct for human-centered and ethical Extended Reality (XR) and how it was operationalised through an Ethical Impact Assessment (EIA) and a Compliance Checklist. The paper is relevant because XR governance currently has many high-level principles and comparatively few concrete instruments that developers and deployers can use from ideation to deployment. This manuscript aims to convert ethical ambition into usable practice.

 Major points

 Translation from empirical evidence to principles to normative claims: The manuscript asserts a move from project deliverables and stakeholder inputs to nine principle domains, seven articles, and operational tools. In order to establish concrete guidelines for action, it is necessary to spell out ethical principles. The ambition is right. The paper does not stop at listing values and principles but offers specific obligations that can foster reflection and inform practice. However, there is always the risk to obscure the translation pathway, i.e. that the reader cannot easily see which project findings generated which principles, which articles, and which questions for the EIA. By providing a comprehensive appendix (Bernabe et al., 2025), this methodological procedure becomes clear. The translation pathway could be improved by disclosure of so-called bridge principles (Kuelmeyer et al, 2022), i.e. explicating the normative principles that bridge empirical findings and normative claims.

 NotebookLM-supported generation of EIA questions: The manuscript states that project deliverables were uploaded to NotebookLM and prompts were used to generate questions for ethically relevant risks under each area of impact. The approach is efficient and fits the translational aim. I do not deem it necessary to make the prompt strategy and outputs from NotebookLM transparent since the outputs underwent an iterative human review and represent just a starting point for the method of reflective equilibrium.

 Reflective equilibrium as a method: The paper states that drafting and revision were guided by reflective equilibrium. This is an appropriate methodological choice for reconciling empirical inputs, normative principles, and practical constraints. However, the manuscript does not show reflective equilibrium in operation. Without an example, the method could be read as a legitimating phrase rather than a documented procedure. If the authors would provide a specific example of a revision and outline the rules for resolving conflicts, this would make the paper even more convincing.

 Participation of different stakeholder groups: The manuscript reports consultations via email, targeted meetings, the XR4human forum, and LinkedIn, with feedback logged weekly and incorporated iteratively. The multi-channel design is well-suited to reach heterogeneous stakeholders and to test clarity and practicality.

 Minor points

 Terminology and scope: Authors could define early what counts as XR in this document, especially with respect to mixed reality, virtual worlds, and metaverse-adjacent systems.

 Domains of application: Authors could give a hint on the different domains of application of their work, e.g. healthcare, industry, entertainment etc.

 Overall recommendation

 The paper is in very good shape; the contribution is timely and practically oriented. The paper could be improved by disclosing some minor methodological issues.

**References**

 Refer to reference no. 1

Where applicable, are recommendations and next steps explained clearly for others to follow? (Please consider whether others in the research community would be able to implement guidelines or recommendations and/or constructively engage in the debate)

Yes

Does the article adequately reference differing views and opinions?

Yes

Are all factual statements correct, and are statements and arguments made adequately supported by citations?

Yes

Is the rationale for the Open Letter provided in sufficient detail? (Please consider whether existing challenges in the field are outlined clearly and whether the purpose of the letter is explained)

Yes

Is the Open Letter written in accessible language? (Please consider whether all subject-specific terms, concepts and abbreviations are explained)

Yes

Reviewer Expertise:

ethical issues of XR in surgery, data ethics, AI ethics, research ethics, digital bioethics, medical ethics, history of medicine

I confirm that I have read this submission and believe that I have an appropriate level of expertise to confirm that it is of an acceptable scientific standard.

## References

[1] : Empirical Research and Recommendations for Moral Action: A Plea for the Transparent Reporting of Bridge Principles in Public Health Research. *Public Health Ethics* .2022;15(2) : 10.1093/phe/phac002147-159 10.1093/phe/phac002

# Response

**Published:** 2026-06-29

**Authors:** Shereen Cox

**Affiliations:** Universitetet i Oslo, Oslo, Oslo, Norway

We thank the reviewer for this thoughtful input and for pointing us to Kuehlmeyer et al. (2022), which we found highly insightful. We agree that making the “translation pathway” between empirical findings and normative recommendations more transparent by distinguishing descriptive claims, bridge principles, and normative conclusions, can substantially strengthen the methodological clarity of empirical ethics work. At the same time, providing a fully explicit mapping for all nine principle domains, seven articles, and all EIA questions would require us to reconstruct, in detail, the reasoning across multiple work‑package deliverables, numerous internal workshops, and stakeholder consultations over several years. This is methodologically valuable but, in our view, not practically feasible within the format and purpose of this Open Letter, which is to give a concise account of the process and resulting tools rather than an exhaustive reconstruction of every inferential step. Kuehlmeyer et al. themselves note that such reconstruction becomes particularly complex in guideline‑like outputs that integrate findings from multiple studies and involve collaborative, multi‑stage deliberation: “guidelines … often integrate findings from multiple studies, their methodology is based on a collaborative process involving experts in a certain field and they include more complex processes of reasoning and deliberation”. This is precisely the situation for the  Code of Conduct and its operationalization.  We have revised to include definition of XR terminologies. This was also included in the glossary of terms of the Code of Conduct. We have included different use cases that may be relevant for the application of the Code of Conduct in the operationalisation and dissemination section. We have added a section on normative reflection and synthesis, explaining with examples how reflective equilibrium was applied to the different impact domains as to a middle ground. We believe this has improved the open letter.

# Reviewer report: Reviewer response for version 1

**DOI:** 10.21956/openreseurope.24064.r69117

**Published:** 2026-02-24

**Authors:** Joshua A Fisher

**Affiliations:** 1 Ball State University, Muncie, Indiana, USA

The authors of this open letter present a pressing issue and their participatory process for meeting the challenge of creating ethical XR experiences. I concur with their work and am generally appreciative of the code of conduct as detailed on the initiative’s website. My suggestions for revisions are meant to clarify the stakes and to integrate some of the diligent work this group has done into the letter. In this draft, this work is pointed to or gestured to, and a more descriptive and, while not analytical, propositional summary might achieve more constructive ends for this letter.

 Within the introduction, I strongly suggest that the authors reference the distinct ethical challenges addressed by XR4Human. Provide readers with examples so that future researchers might have a better understanding of the ethical stakes of immersive technologies. I would not cut any of the discussion of the process or the goal, but I would add more details to the initiative. Please cite the key peer-reviewed publications and/or flagship deliverables that directly informed the Code, rather than only pointing generally to the website. This information enhances the project's credibility. The authors might also expand on or place Wright’s work in conversation with industry groups such as XRSI and with academic white papers from IEEE. I expect such discussions occurred in XR4Human’s process for developing their Code, and elevating them to this letter strengthens the perception of deliberation and multiperspectivity, crucial to ethics and technology.

 The approach section could be greatly strengthened by providing more details and examples of the deliberations that occurred. Future researchers in the space would best learn by a guiding example that demonstrates how brainstorming and stakeholder consultations proceed. Were any design methods used? What approaches to ethics were explored? Who were the groups that were most present in deliberations (industry, nationality, etc.)? What kinds of engagement did the researchers get on LinkedIn? Please report the scale and composition of consultation (approximate N, stakeholder categories, countries/sectors where feasible) and describe at a high level how feedback was categorized and incorporated.  How did they review that information? How was it dismissed or integrated? As a reader, I am left with more questions than answers. Providing some of this information strengthens the perception of the approach and enables future researchers to replicate it.

 In the synthesis section, since the researchers utilized NotebookLM, it would be beneficial to know which Gemini model was used. Further, what prompts were used and during what time period? Were they the same prompts from information gathering? How were humans involved in the loop? What information verification protocols were implemented? How were the weekly meetings run? It is good to see deliberation, but what was the standard for consensus?

 Each principle, article, and key impact area should include at least a short definition and an example. This will help researchers pick up the authors’ work and continue.

 While I appreciate that the infographic is meant to motivate readers to engage with the XR4HUMAN site, the authors might consider adding XR4HUMAN’s *Human-Centered and Ethical Development of Immersive Technologies Checklist*, as it is more directly actionable. Alternatively, the authors could include a graphic that helps readers identify common categories of ethical issues and points them to the relevant parts of the Code of Conduct on the site.

 I recommend Approved with Reservations. The initiative and outputs are timely and potentially valuable for XR researchers and practitioners; however, the Open Letter currently reads more as a pointer to external resources than a self-contained scholarly communication. Adding concrete examples of the ethical stakes addressed, minimal reporting of the consultation process (who/roughly how many and how feedback was synthesized), and transparency about the NotebookLM-supported drafting workflow would substantially strengthen credibility, reuse, and replicability.

Where applicable, are recommendations and next steps explained clearly for others to follow? (Please consider whether others in the research community would be able to implement guidelines or recommendations and/or constructively engage in the debate)

Partly

Does the article adequately reference differing views and opinions?

No

Are all factual statements correct, and are statements and arguments made adequately supported by citations?

Yes

Is the rationale for the Open Letter provided in sufficient detail? (Please consider whether existing challenges in the field are outlined clearly and whether the purpose of the letter is explained)

Partly

Is the Open Letter written in accessible language? (Please consider whether all subject-specific terms, concepts and abbreviations are explained)

Yes

Reviewer Expertise:

XR UX, XR design and development, XR Interactive Storytelling, and XR Ethics

I confirm that I have read this submission and believe that I have an appropriate level of expertise to confirm that it is of an acceptable scientific standard, however I have significant reservations, as outlined above.

# Response

**Published:** 2026-06-29

**Authors:** Shereen Cox

**Affiliations:** Universitetet i Oslo, Oslo, Oslo, Norway

We thank the reviewer for the detailed, relevant and constructive feedback.

 The intent was to introduce the Code of Conduct and give an overview of the process, hence the choice of an open letter. However, the reviewer’s comments and interest in the credibility, transparency of the process are well received, and changes made to reflect this. The introduction section has been expanded to elaborate on some of the main ethical issues of XR especially the key findings of WP2 deliverables. This is an attempt to address the reviewer’s comments on clarifying the stakes. We also note some of the legal implications and risks of XR.  The XR4Human Code of Conduct was developed using a participatory co-design methodology facilitated through expert-led workshops as well as stakeholder and public consultations. In the approach section, we have now included the different professional expertise of the working group, how brainstorming and stakeholder consultations were conducted. The design method is participatory co-design while applying reflective equilibrium for identification and synthesis of principles.  

 LinkedIn and XR4Human forum were used strictly as stakeholder engagement and dissemination platforms. The draft Code of Conduct was posted, feedback invited by way of comments and direct feedback on draft materials, which were logged, reviewed during weekly meetings, and considered during iterative document revisions. The methods section has been updated to include the process of integration, the stakeholder/industry categories/countries and sectors.  A master prompt was inputted into  Google Notebook LM  (Gemini  2.5 Flash). It was only used for the initial drafting of questions relevant to areas of impact. Thereafter no LM was involved and only human review, refinement and finalisation. This was different from the prompts used in the consultative phase.  

 Two researchers did the initial drafting of impact descriptors. This was done over several months in weekly meetings. The draft EIA and Compliance checklist were shared with four members of the consortium (WP on interoperability and WP on Experience Library) for feedback and refinement before submission to the working group. Consensus was achieved when there was no more disagreement among team members.  

 A section has been included with a summary of the five areas of impact, the relevant principles and articles. These have been defined or elaborated further to facilitate better understanding by the reader.  

 With regard to the graphic, we thank the reviewer for this suggestion. Please note, we reflected on the implications of using a risk-based framing, as done in one of our previous papers and decided that placing emphasis on areas of impact rather than risks would be more constructive and accessible for both developers and end-users, while still capturing the potential ethical concerns associated with XR technologies. The impact graphic is therefore included in the Ethical Impact Assessment.
